# Multimodal melphalan delivery: improved safety and outcomes with autologous myeloma transplant in India

**DOI:** 10.5588/pha.26.0046

**Published:** 2026-08-24

**Authors:** A. Joy, A. Philip, R. Muralikrishnan, K. Niraimathi, R. Zachariah, P. Thekkur, A. Doshi, M.P. Arya, K. Ajith, V. Hari, V. Rajan, M.O. Lissy, R. Chandran, V.A. Anoop, L. Abraham, P.S. Alwyn, A.A. Alex, S. Cyriac

**Affiliations:** 1 Department of Medical Oncology, Rajagiri Hospital, Kochi, India;; 2 Evidentia Research Solutions, Chennai, India;; 3 UNICEF, UNDP, World Bank, WHO Special Programme for Research and Training in Tropical Diseases (TDR), Geneva, Switzerland;; 4 Centre for Operational Research, International Union against Tuberculosis and Lung Disease, Paris, France;; 5 Department of Nursing, Rajagiri Hospital, Kochi, India;; 6 Department of Transfusion Medicine, Rajagiri Hospital, Kochi, India;; 7 Department of Pathology, Rajagiri Hospital, Kochi, India;; 8 Department of Pharmacology, Rajagiri Hospital, Kochi, India;; 9 Department of Advanced flow cytometry, Rajagiri Hospital, Kochi, India.

**Keywords:** universal health coverage, SORT-IT, SDG, operational research

## Abstract

**BACKGROUND:**

High-dose melphalan–based autologous stem cell transplantation (ASCT) remains a cornerstone of treatment in multiple myeloma but causes severe gastrointestinal toxicity in up to 50% of patients due to mucosal injury.

**OBJECTIVE:**

To mitigate this, we assessed a multimodal approach of combined oral cryotherapy, extended duration of melphalan infusion (1 h compared to bolus), and upright patient positioning during administration.

**DESIGN:**

A cohort study using hospital patient records to evaluate the safety and outcomes of this approach at a tertiary hospital in South India, from January 2017 to March 2026.

**RESULTS:**

Among 47 patients undergoing ASCT, 4% had grade 3 (severe) mucositis, 15% experienced grade 3 diarrhoea, and one patient had severe vomiting. No grade 4 (life threatening) or grade 5 (fatal) gastrointestinal toxicities were observed. Total parenteral nutrition was required in 4% patients. The median duration of stay in the transplant unit was 14 days. There was no post-transplant mortality within 1 year. Relapse-free survival at 1 year was 88%, and relapse was significantly associated with high-risk cytogenetics.

**CONCLUSION:**

Multimodal melphalan delivery is feasible, with manageable toxicity and low rates of severe adverse events, while maintaining excellent short-term outcomes. Further multicentric evaluation and wider adoption is warranted.

## Graphical abstract

**Figure:**
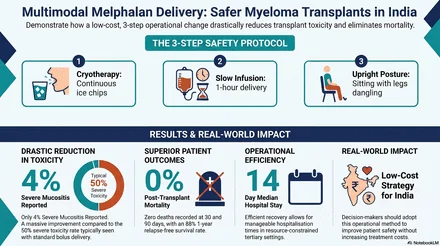

Multiple myeloma is a cancer of plasma cells; a type of white blood cells found in the bone marrow. The uncontrolled proliferation of these cells along with disruption of normal blood cell production leads to complications such as anaemia, bone damage, kidney dysfunction, hypercalcaemia, and increased susceptibility to infections. The management of multiple myeloma starts with an induction phase, where a combination of drugs is used to reduce tumour burden. Eligible patients, then receive high-dose melphalan (HDM) to eliminate residual cancer cells, followed by autologous stem cell transplantation (ASCT) to restore bone marrow function. This approach is the standard of care and has been shown to increase disease-free survival.^1–4^ Despite its therapeutic efficacy, HDM is associated with significant toxicity, notably gastrointestinal adverse events such as oral mucositis, nausea, vomiting, and diarrhoea.^3,4^ These adverse events often lead to prolonged hospitalisation, increased reliance on supportive medication including analgesics, the need for parenteral nutrition, electrolytes, and an elevated risk of infection.^5–7^

Operational strategies to mitigate HDM-related toxicity are needed to enhance quality of care, improve patient safety, and reduce costs both for patients and health systems. In this vein, three supportive interventions merit attention. First, oral mucositis is one of the most common dose-limiting toxicities of HDM with severe grades occurring in more than 50% of patients when melphalan is given at a dose of 200 mg/m^2^.^1^ The pathophysiology involves direct cytotoxic injury to the basal epithelial layer, compounded by inflammation and secondary infection.^8^ Oral cryotherapy during HDM administration induces local vasoconstriction, thereby reducing mucosal drug exposure. This intervention is currently recommended as it has been shown to significantly reduce both the incidence and severity of mucositis.^9–12^ Second, melphalan’s short terminal half-life (approximately 1.5 h) suggests that infusion duration may influence toxicity. Rapid bolus infusions (given within 15–20 min) lead to higher peak plasma concentrations and greater mucosal exposure, while prolonged infusions may attenuate mucosal injury by reducing peak concentrations and enhancing systemic drug clearance. Emerging evidence suggests that extended infusion durations may reduce gastrointestinal toxicity without compromising therapeutic efficacy.^13,14^ However, further real-world studies are needed to better understand the clinical implications of this approach in routine settings. Finally, upright patient positioning may influence splanchnic (mesenteric) blood pooling which has been recognised as a potential modulator of gastrointestinal drug distribution and mucosal exposure.^15^ Splanchnic blood flow is reduced in the upright position due to gravitational redistribution of blood volume.^16^ Thus, positioning patients in a sitting posture with legs hanging may reduce splanchnic blood flow, thereby limiting melphalan distribution to gastrointestinal mucosa, and subsequently reducing treatment-associated toxicity.

In light of these considerations, since 2017, we have been implementing a multimodal melphalan administration approach incorporating three supportive interventions: oral cryotherapy, extended infusion duration, and upright patient positioning. We hypothesised that this approach could reduce HDM-related toxicity without compromising the regimen’s antineoplastic efficacy. The available data provided an opportunity to assess its real-world impact. Thus, among all multiple myeloma patients admitted to a tertiary hospital in South India and who received melphalan via the multimodal approach prior to ASCT, our specific objectives were to: 1) Describe their demographic and clinical characteristics; 2) Assess the prevalence of gastrointestinal adverse events and complications of HDM during the period of hospital admission; and 3) Determine hospital exit outcomes, the 30 and 90 day post-transplant mortality and relapse-free survival.

## METHODS

### Study design

This was a cohort study using secondary data from hospital electronic medical records.

### General setting

India is the world’s most populous country, and it has a mixed health care system comprising of both public and private providers. Access to tertiary care for complex diseases like cancer faces significant geographic and socio-economic disparities. Multiple myeloma accounts for approximately 1% of all cancers and 10% of haematologic malignancies in India with an estimated incidence of approximately 1 per 100,000 population.^17,18^ Access to standard-of-care therapies like ASCT is limited to urban tertiary care centres and financially prohibitive for many patients. While Regional Cancer Centres and government cancer hospitals offer subsidised care, they are overburdened with long patient waiting times.^19,20^

### Specific setting

The Rajagiri tertiary hospital was the study site. It is a 500-bed, multi-specialty tertiary care facility located in Kochi, Kerala, South India, and is certified by the National Accreditation Board for Hospitals & Healthcare Providers for quality of care. There are departments of medical oncology and haematology providing chemotherapy, immunotherapy, targeted therapy, and haematopoietic stem cell transplantation. There is also a dedicated bone marrow transplant (BMT) unit with specialist teams and trained nursing staff. The unit is also supported by infectious disease specialists, dieticians, and transfusion medicine specialists. ASCT is offered for multiple myeloma with protocols aligned with international guidelines.^21^ ASCT is fee-based (6,000–8,000 USD), but the hospital participates in insurance programmes and philanthropic initiatives to partially subsidise care for financially underprivileged patients.

### Autologous stem cell transplant procedure

All eligible myeloma patients who have achieved at least a partial response to induction therapy and deemed fit after pre-transplant workup are admitted in the Medical Oncology Department for ASCT after informed consent. The eligibility criteria are shown in Table 1 and the protocol timeline for ASCT is given in Table 2. All eligible patients should proceed to ASCT immediately after successful induction therapy to maximise the likelihood of progression-free survival.^4,22,23^ Despite counselling, patient uptake of ASCT may be influenced by cost considerations, fear of side effects and death, and logistic considerations. The initial phase consists of stem cell mobilisation which includes administration of granulocyte colony stimulating factor (G-CSF) given subcutaneously at a dose of 10 mcg/kg daily in two divided doses for 4–5 days prior to stem cell collection for transplant. In patients with low peripheral blood stem cell counts, plerixafor is administered subcutaneously at a dose of 0.24 mg/kg approximately 11 h prior to aphaeresis. Stem cells are harvested from peripheral blood by aphaeresis procedure prior to transplant. If the stem cell harvest is adequate (i.e., >2 × 10^6^ CD34^+^ cells/kg), the patient is transferred to the BMT unit. This is followed by infusion of HDM (140–200 mg/m^2^) after standard premedication and hydration using the multimodal administration method.

**TABLE 1.** Transplant eligibility criteria for multiple myeloma in Rajagiri Hospital, India (2017–2026).

Criterion	Eligibility criteria
Age	≤70–75 years (physiologic age more important than chronologic age)
Performance status	ECOG PS 0–2 or Karnofsky ≥70%
Disease status	Patients should achieve at least a partial response, very good partial response, or complete response to induction therapy
Cardiac function	Left ventricular ejection fraction ≥40%
Pulmonary function	DLCO, FEV1, FVC ≥ 50% of predicted
Renal function	Creatinine clearance ≥30 mL/min (dose-adjustment of melphalan is required if < 30 mL/min)
Hepatic function	Transaminases < 3× ULN, bilirubin <2 mg/dL
Infectious disease	No active uncontrolled infections
Haematologic status	Ability to mobilise sufficient peripheral blood stem cells (≥2 × 10^6^ CD34^+^ cells/kg) for ASCT
Psychosocial status	Ability to provide informed consent and having adequate social support.
Comorbidities	No uncontrolled comorbidities that would increase transplant-related mortality – Charlson Comorbidity Index guides assessment.

ECOG PS = Eastern Cooperative Oncology Group Performance Status; DLCO = diffusion limit of carbon monoxide; FEV1 = forced expiratory volume in 1 second; FVC = forced vital capacity; ULN = upper limit of normal; ASCT = autologous stem cell transplantation.

**TABLE 2.** Protocol timeline for autologous stem cell transplant for myeloma at Rajagiri Hospital, India (2017–2026).

Day of transplant	Planned interventions
Day −5	Injection G-CSF 5 mcg s/c BD
Day −4	Injection G-CSF 5 mcg s/c BD
Day −3	Injection G-CSF 5 mcg s/c BD
Day −2	Injection G-CSF 5 mcg s/c BD
	Central line insertion
	Femoral sheath insertion for aphaeresis
	Injection Plerixafor 0.24 mg/kg given 11 h prior to aphaeresis
Day −1 (one day prior to transplant)	Stem cell collection via aphaeresis procedure
	Proceed to administer high-dose melphalan (140–200 mg/m^2^) if stem cell collection is adequate (i.e., >2 × 10^6^ CD34 cells/kg)
Day 0 (transplant day)	Stem cell infusion
Day +3	Administer pegylated G-CSF 6 mg s/c

Days are calculated relative to the day of stem cell infusion, which is designated as Day 0. Days prior to stem cell infusion are indicated with negative numbers and days post-infusion are indicated with positive numbers.

G-CSF = granulocyte colony stimulating factor; s/c = subcutaneous route; BD = bis in die (twice daily).

### Multimodal administration of high-dose melphalan for ASCT

Melphalan is administered sequentially, with each 50 mg vial reconstituted individually and infused intravenously over 10 min using normal saline to achieve the target total dose (140–200 mg/m^2^) with an extended duration of approximately 1 h. Oral cryotherapy using ice chips is performed throughout the infusion to mitigate the risk of oral mucositis. Additionally, patients are positioned sitting with legs dangling by the side of the bed during the infusion. Incident toxicities are graded using the Common Terminology Criteria for Adverse Events (version 5).^24^

Stem cells are re-infused after 24 h of melphalan infusion on the day of transplant. Pegylated G-CSF is given on the third day post-transplant as a single dose to hasten engraftment. Patients are discharged from the BMT unit after successful engraftment and resolution of acute toxicities. The patients are reviewed as out-patients and assessed for clinical follow-up after 2 weeks of discharge. Maintenance therapy is typically instituted after 1 month of ASCT depending on the clinical status of the patient. Routine clinical follow-up visits are then scheduled monthly, with disease status evaluation conducted every three months.

### Study population and period

This included all consecutive multiple myeloma patients who underwent ASCT following multimodal melphalan administration between January 2017 and March 2026.

### Data variables and validation

All data variables were extracted from electronic medical records and cross-checked with individual patient records by the principal investigator and two other data experts from the SORT-IT data team. Validated data was entered into an electronic data collection pro forma and transferred into Microsoft excel spreadsheet. Data variables included demographic and clinical characteristics, dates of admission and discharge, date of melphalan administration and stem cell infusion, performance status, cytogenetic risk, International Staging System for myeloma, type and grade of toxicities, complications, and hospital exit outcomes.

### Statistical analysis

Data were analysed using Stata (Stata Corp LLC: Release 14). We used descriptive statistics to summarise clinicodemographic data and grade of adverse events. Continuous variables like melphalan dose, stem cell doses, and length of stay in the BMT unit were reported as median with range. Hospital exit outcomes and post-transplant mortality at 30 days and 90 days was reported. The Kaplan–Meier method was used to graphically present the relapse-free survival. The comparison of adverse events and relapse with clinicodemographic parameters was done using the χ² test, and a *P* value of ≤0.05 was considered significant.

### Ethical statement

Ethics approval was obtained from the Rajagiri Hospital Ethics and Scientific Review Committee (No: RAJH/2025/139, dated 28 July 2025). As the study involved a review of patient records with no personal identifiers, a waiver for informed consent was granted by the ethics committees.

## RESULTS

A total of 47 patients with multiple myeloma (mean age 56 years; 51% women) underwent ASCT following HDM. The baseline demographic and clinical characteristics are presented in Table 3. Most patients (92%) had good functional status (ECOG 1) and 77% presented at earlier stages of disease (stages I and II). Comorbidities were present in 75% of patients. Based on cytogenetic classification, 51% had standard-risk disease, 21% had high-risk disease, and 28% had unknown cytogenetic risk.

**TABLE 3.** Demographic and clinical characteristics of patients who received melphalan via multimodal administration prior to undergoing autologous stem cell transplant at Rajagiri Hospital, India (2017–2026).

Characteristics	N (%), total = 47
Demographic characteristics	
Age, years (mean, SD)	55.7 ± 7.0
Sex	
Male	23 (48.9)
Female	24 (51.1)
Clinical characteristics	
BMI^A^ (kg/m^2^)	
Underweight	0
Normal weight	21 (44.7)
Overweight	21 (44.7)
Obese	5 (10.6)
ECOG^B^ performance status	
0	3 (6.4)
1	43 (91.5)
2	1 (2.1)
Presence of comorbidities	
Yes	35 (74.5)
No	12 (25.5)
Number of comorbidities (n = 35)	
1	19 (54.3)
2	10 (28.6)
3	5 (14.3)
>3	1 (2.8)
Cytogenetic risk	
High risk	10 (21.3)
Standard risk	24 (51)
Unknown	13 (27.7)
International staging system stage	
Stage 1	21 (44.7)
Stage 2	15 (31.9)
Stage 3	11 (23.4)
Presenting symptoms (CRAB)^C^	
Hypercalcaemia	7 (14.9)
Renal impairment	11 (23.4)
Anaemia	22 (46.8)
Bone lesions	46 (97.9)
Induction regimen prior to transplant	
VRD^D^-based regimen	31 (66.0)
VCD^E^-based regimen	8 (17.0)
Other regimens	8 (17.0)
Daratumumab used during induction	2 (4.3)
Prior lines of induction therapy	
1	36 (76.6)
2	9 (19.1)
>2	2 (4.3)
Disease status pre-transplant	
Complete remission	32 (68.1)
Very good partial response	14 (29.8)
Partial response	1 (2.1)
Plerixafor used for stem cell mobilisation	36 (76.6)
Melphalan dose reduction for CKD^F^	5 (10.6)
Melphalan dose (mg/m^2^) (median, range)	180 (140–200)
Stem cell dose infused (CD34^+^ cells/kg) (median, range)	7.0 × 10^6^ (1.6–8.0 × 10^6^)

A Body mass index.

B Eastern Cooperative Oncology Group.

C Hypercalcaemia, renal failure, anaemia, bone lesions.

D Bortezomib, lenalidomide, dexamethasone.

E Bortezomib, cyclophosphamide, dexamethasone.

F Chronic kidney disease.

The most common presenting features were bone lesions (98%), followed by anaemia (47%). Most patients received a VRD regimen (bortezomib, lenalidomide, dexamethasone) for induction therapy (66%), while 17% received VCD regimen (bortezomib, cyclophosphamide, dexamethasone). Only two patients (4%) received daratumumab-containing therapy. Prior to ASCT, most patients achieved a good response to induction therapy, with 68% achieving complete remission and 30% achieving very good partial response.

### Prevalence of gastrointestinal adverse events and complications

Of the 47 patients in the study, grade 1 and grade 2 oral mucositis occurred in 91% and only 4% had severe grade 3 toxicity (Table 4). Low-grade diarrhoea occurred in 60% with only 15% experiencing severe grade toxicity. Emesis occurred in 64% of patients, primarily low grade. No grade 4 or grade 5 gastrointestinal toxicities were observed. Among treatment-related complications, febrile neutropenia occurred in 47% of patients, bleeding events in 13%, and septicaemia in 9%. Supportive care interventions were common; 81% of patients required correction of electrolyte imbalances, 66% required intravenous antibiotics, and only 4% required total parenteral nutrition.

**TABLE 4.** Adverse events, complications, and supportive management of high-dose melphalan infusion at Rajagiri Hospital, India (2017–2026).

CTCAE grade^A^ (adverse events)	Description	N (%)
Oral mucositis		
Grade 0	No toxicity	2 (4.3)
Grade 1	Asymptomatic or mild symptoms; erythema of mucosa	26 (55.3)
Grade 2	Moderate pain or ulcer that does not interfere with oral intake, modified diet indicated	17 (36.1)
Grade 3	Severe pain; interferes with oral intake	2 (4.3)
Diarrhoea		
Grade 0	No toxicity	12 (25.5)
Grade 1	Increase of <4 stools/day over baseline; not interfering with ADL	12 (25.5)
Grade 2	Increase of 4–6 stools/day over baseline, interfering with ADL	16 (34.1)
Grade 3	Increase of ≥7 stools/day over baseline; incontinence; hospitalisation indicated; severe interference with ADL	7 (14.9)
Vomiting		
Grade 0	No toxicity	17 (36.2)
Grade 1	Mild nausea or 1–2 episodes in 24 h	20 (42.6)
Grade 2	3–5 episodes in 24 h requiring intervention	9 (19.1)
Grade 3	≥6 episodes in 24 h requiring hospitalisation	1 (2.1)
Complications		
	Febrile neutropenia	22 (46.8)
	Bleeding events	6 (12.8)
	Septicaemia (positive blood culture)	4 (8.5)
Management		
	Electrolyte imbalance requiring correction	38 (80.9)
	Intravenous antibiotics requirement	31 (66.0)
	Total parenteral nutrition requirement	2 (4.3)
	Analgesia requirement (>/ = WHO Step 2)	2 (4.3)

CTCAE = Common Terminology Criteria for Adverse Events (version 5); ADL = activities of daily living.

A Grade 4 events (life threatening toxicity) and Grade 5 events (death) were not observed in the study population.

### Haematological recovery and hospitalisation time

The absolute neutrophil count was under 500 cells/µL (nadir) on the 5th day (median) after transplant (range 3–7). The median duration of neutropenia was 4 days (range 2–6). Neutrophil engraftment occurred at a median of 9 days (range 8–10), while platelet engraftment was achieved at a median of 10 days post-transplant (range 8–15). Packed red blood cell transfusions were required in 21% of patients. The median single donor platelet requirement was one unit per patient (range 0–2.5). The median duration of stay in the BMT unit for transplantation was 14 days (range 12–19).

### Hospital exit and survival outcomes

All patients were discharged alive following transplantation and there were no deaths at either 30 days or 90 days post-transplant. During follow-up, there were seven relapses including five within 1 year and two thereafter (see Figure). These relapses occurred in four patients with higher-risk cytogenetics, one patient with standard risk, and two without baseline cytogenetic evaluation. There was a significant association of high-risk cytogenetics with relapse (*P* < 0.05). Relapse-free survival at 1 year post-transplant was 88%. Among the 43 patients who had completed at least 1 year of follow-up, there were four deaths occurring between 15 and 49 months post-transplant.

**FIGURE.**
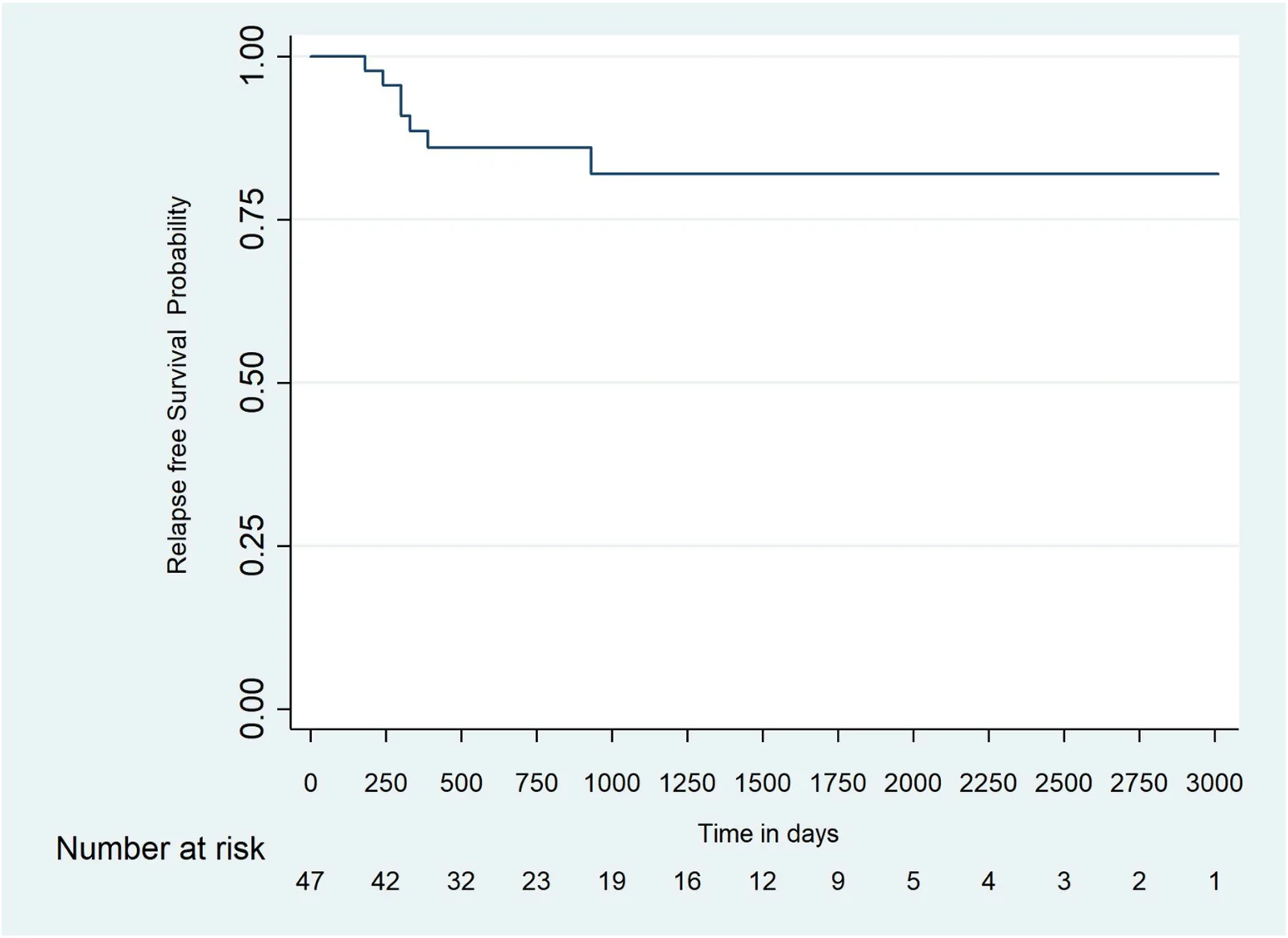
Relapse-free survival probability for multiple myeloma patients who underwent autologous stem cell transplant at Rajagiri Hospital, India (2017–2026).

## DISCUSSION

This is the first study from South India, and it shows that HDM, combined with multimodal supportive interventions, can be delivered safely with manageable toxicity and excellent short-term outcomes. Oral cryotherapy, extended melphalan infusion duration, and upright positioning were associated with manageable gastrointestinal toxicity with most adverse events being low grade. There was no transplant-related mortality at 30 or 90 days post-transplant, and all patients were discharged after a median stay of 14 days.

ASCT remains the cornerstone for consolidation of response in eligible myeloma patients. No deaths and manageable toxicity highlight operational feasibility and safety of this approach in resource-constrained environments. These findings would also provide reassurance to clinicians in our setting who through clinical observation believed the multimodal infusion approach is beneficial.

The study has several strengths. These include use of real-world clinical data, enhancing generalisability to routine practice; data were systematically cross-validated enhancing reliability; standardised criteria were used for staging and reporting adverse events to ensure consistent reporting; and comprehensive clinical and outcome data were available. The study also adhered to Strengthening the Reporting of Observational Studies in Epidemiology (STROBE) guidelines on the reporting of observational studies in epidemiology.^25^ There are also some limitations. The sample size was relatively small (n = 47), especially considering that recruitment occurred over a period exceeding 9 years (2017–2026), limiting statistical power and subgroup analyses. Cytogenetic risk data were unavailable for 28% of patients, potentially compromising our estimation of relapse risk. Follow-up period was short, limiting assessment of longer-term outcomes. Finally, absence of a comparator arm (e.g., bolus melphalan) limited attribution of causal benefit to the multimodal approach. Therefore, findings should be interpreted cautiously and considered hypothesis-generating and larger multicentre studies with longer follow-up are warranted.

There are several policy and practice implications. First, diarrhoea was mostly low grade; only 14% had grade 3 and none had grades 4–5. This figure is about threefold lower than reported elsewhere (40%–50%).^2,5,7^ This welcome finding could be attributed to the combination of prolonged melphalan infusion and reduced splanchnic blood pooling from upright positioning which favourably influenced drug distribution and consequent intestinal mucosal injury. However, pharmacokinetic studies would be needed to substantiate these observations which could reduce the morbidity and costs associated with ASCT. Second, 47% developed febrile neutropenia and about 81% had electrolyte imbalances, underscoring the need for vigilant monitoring and well-trained teams to ensure high standards for infection control, prompt administration of antibiotics, and meticulous electrolyte correction. Third, most post-transplant relapses occurred within 1 year and in high cytogenetic risk patients. Increasing access to daratumumab (CD38 monoclonal antibody) during induction may improve progression-free survival,^26,27^ but cost is a major barrier (approximately USD 40,000 per year only for the drug). In our cohort, only two patients received it. We advocate for urgent access to affordable Biosimilar formulations of the drug. Finally, about 25% of eligible patients did not undergo transplantation immediately after first remission, potentially compromising progression-free survival.^3,4,21–23^ Strengthening patient education, counselling, and prompt referrals are essential to ensure early uptake of ASCT.

## CONCLUSION

Our study demonstrates that it is feasible to offer HDM and ASCT with multimodal supportive interventions. Toxicity was manageable and short-term outcomes were favourable. This implementation research introduces an operational drug delivery that may reduce complications and we advocate for further evaluation of this approach.
